# *In Vitro* Antibacterial Activity of Unconjugated and Conjugated Bile Salts on *Staphylococcus aureus*

**DOI:** 10.3389/fmicb.2017.01581

**Published:** 2017-08-23

**Authors:** Thippeswamy H. Sannasiddappa, Peter A. Lund, Simon R. Clarke

**Affiliations:** ^1^School of Biological Sciences, University of Reading Reading, United Kingdom; ^2^Institute of Microbiology and Infection, School of Biosciences, University of Birmingham Birmingham, United Kingdom

**Keywords:** viability, intracellular pH, membrane permeability, bile salts, antibacterial

## Abstract

Bile salts are potent antimicrobial agents and are an important component of innate defenses in the intestine, giving protection against invasive organisms. They play an important role in determining microbial ecology of the intestine and alterations in their levels can lead to increased colonization by pathogens. We have previously demonstrated survival of the opportunistic pathogen *Staphylococcus aureus* in the human colonic model. Thus investigating the interaction between *S. aureus* and bile salts is an important factor in understanding its ability to colonize in the host intestine. Harnessing bile salts may also give a new avenue to explore in the development of therapeutic strategies to control drug resistant bacteria. Despite this importance, the antibacterial activity of bile salts on *S. aureus* is poorly understood. In this study, we investigated the antibacterial effects of the major unconjugated and conjugated bile salts on *S. aureus*. Several concentration-dependent antibacterial mechanisms were found. Unconjugated bile salts at their minimum inhibitory concentration (cholic and deoxycholic acid at 20 and 1 mM, respectively) killed *S. aureus*, and this was associated with increased membrane disruption and leakage of cellular contents. Unconjugated bile salts (cholic and deoxycholic acid at 8 and 0.4 mM, respectively) and conjugated bile salts (glycocholic and taurocholic acid at 20 mM) at their sub inhibitory concentrations were still able to inhibit growth through disruption of the proton motive force and increased membrane permeability. We also demonstrated that unconjugated bile salts possess more potent antibacterial action on *S. aureus* than conjugated bile salts.

## Introduction

The human intestine is a complex ecosystem composed of commensal microflora species, various types of secretory fluids, fermentation metabolites of digested food, and host defense molecules (Sekirov and Finlay, 2009). A broad range of innate defenses in the intestine include acidic pH, fermentation metabolites like short chain fatty acids (SCFA), high osmolarity, local gut mucosal immunity, colonization resistance by normal commensal bacteria, and bile salts. Despite these properties, the intestine is host to a complex microflora (Quigley, 2013; Zhang et al., 2015). While many of these microorganisms live as harmless commensals, there are also opportunist pathogens (Baumler and Sperandio, 2016). Survival and subsequent colonization by opportunist pathogens of the human intestinal tract requires them to resist these innate defenses. Thus study of innate antimicrobials may lead to development of novel control strategies against pathogens.

*Staphylococcus aureus* is a highly adaptable human opportunistic pathogen responsible for many infections and deaths worldwide (Kluytmans and Wertheim, 2005; Tong et al., 2015). The bacterium lives as a commensal in the nares of 20–25% of the population at any one time (Peacock et al., 2001; Wertheim et al., 2004). While nasal colonization is a well-established risk factor for most types of *S. aureus* infections, several studies have suggested that intestinal colonization, which occurs in 20% of individuals, could have important clinical implications (Acton et al., 2009). Patients with *S. aureus* intestinal colonization can serve as an important source of transmission as they often contaminate the adjacent environment (Masaki et al., 2003; Kernbauer et al., 2015) and also display an increased frequency of skin colonization (Bhalla et al., 2007; Lindberg et al., 2011). A study in an intensive care unit showed that patients with both rectal and nares colonization by *S. aureus* had a higher risk of disease than did patients with nasal colonization alone (Squier et al., 2002; Faden et al., 2010).

The ability of *S. aureus* to survive in the intestine is an important aspect of its capacity to transmit from host to host. In particular, bile salts in the intestine are important innate defense molecules and potent antibacterial agents (Sung et al., 1993; Itoh et al., 1999; Begley et al., 2005). This may have been part of the basis for the success of a 1000-year-old Anglo-Saxon remedy used to treat microbial eye infections, which contains bile salts and was effective against *S. aureus* infections in chronic mouse wound models (Harrison et al., 2015). Patients with cirrhosis show increased bacterial overgrowth due to decreased levels of intraluminal bile salts in the small intestine (Chang et al., 1998; Bauer et al., 2001) and oral treatment with bile salts in cirrhotic rats resulted in reduced small intestinal bacterial overgrowth (Lorenzo-Zuniga et al., 2003). Thus bile salts serve not only as digestive molecules aiding in lipid digestion, but also as potent antibacterial agents in the intestines (Begley et al., 2005).

Thus the interaction between *S. aureus* and human bile salts is an important factor in its ability to colonize in the host intestine particularly in the colon. Despite this importance, the mode of action of these bile salts on *S. aureus* is still not fully understood. The amphipathic nature and presence of a steroid nucleus in their structure makes human bile salts potent antibacterial agents mainly due to their membrane damaging effects (Scholmerich et al., 1984). Bile salts also inhibit bioenergetic process by intracellular acidification, dissipation of the proton motive force, and induction of DNA damage and protein denaturation (Prieto et al., 2004; Kurdi et al., 2006; Merritt and Donaldson, 2009). In this study, we aimed to determine the mechanism of antibacterial action of major human bile salts on *S. aureus*. This revealed multiple mechanisms of killing of *S. aureus* by the ability of bile salts to disrupt essential membrane functions and bioenergetic processes.

## Materials and Methods

### Bacteria and Growth Conditions

*Staphylococcus aureus* SH1000 (Horsburgh et al., 2002) was grown on Brain Heart Infusion (BHI) (Oxoid) broth or agar at 37°C. All experiments were performed at pH 7 in growth media or buffer as indicated in experimental methods.

### Determination of MIC

Bile salts were purchased from Sigma-Aldrich, United Kingdom. Minimum inhibitory concentrations (MICs) were determined by the broth dilution method as described previously (Sannasiddappa et al., 2015). Briefly, two fold dilutions of bile salts in BHI broth were inoculated with *S. aureus* SH1000 at cell density of 10^6^ CFU/ml. Broth dilution tubes with no visible growth after 16 h incubation at 37°C were read as MIC value. The MIC range determined by two fold serial dilution was further narrowed down to determine appropriate MICs.

### Time-Course Measurement of Bacterial Viability upon Exposure to Bile Salts

Bacterial viability upon exposure to bile salts at different time periods was determined as previously described (Sannasiddappa et al., 2015). Briefly, overnight grown *S. aureus* SH1000 were cultured until mid-exponential phase in fresh BHI broth at 37°C shaking incubator under aerobic conditions. After harvesting, the cells were washed twice in sterile 5 mM HEPES buffer (pH 7.2) with 10 mM glucose and resuspended in the same buffer to an OD_600_ of 0.5. Portions of this cell suspension were incubated with various concentrations of CA, DCA, GCA, and TCA for 30 min at 37°C. At 10-min intervals until 30 min, cell suspension dilutions, from each of the bile salt treated groups, were made with sterile peptone saline diluent. Cells from appropriate dilutions were plated onto tryptic soy agar plates, and incubated overnight at 37°C. Colonies were counted and the Log_10_ CFU/ml viabilities were calculated based on the counts from initial untreated (control) cell suspension.

### Intracellular pH Measurement upon Exposure to Bile Salts

The internal pH of the *S. aureus* SH1000 cells upon exposure to various concentrations of the selected bile salts over a time-course were measured according to the method described previously (Breeuwer et al., 1996) except that the experiments were performed in 50 mM potassium phosphate buffer with pH 7 and 10^7^ CFU/ml cells were preloaded with 3.4 μM of membrane permeable pH sensitive precursor probe 5 (and 6-)-carboxyfluorescein diacetate succinimidyl ester for 15 min at 37°C.

### Measurement of the Transmembrane Electrical Potential

Changes in the membrane potentials of the *S. aureus* SH1000 upon energization and addition of various concentrations of selected bile salts were monitored using cationic membrane potential sensitive cyanine fluorescent dye DiSC_3_ (5), as described previously (Cheng et al., 2009) except that experiments were performed in 5 mM HEPES buffer at pH 7 and the cells were loaded with 1 μM DiSC_3_ (5). Membrane potential measurements were compared with 4 μM valinomycin positive control.

### Determination of Intracellular Potassium Levels

The intracellular pool of potassium upon treatment with various concentrations of selected bile salts was quantified by atomic absorption spectroscopy as described previously (Ioannou et al., 2007) except that experiments were performed in 5 mM HEPES buffer (pH 7) at a cell density of 10^8^ CFU/ml. Samples from each of the test (bile salts) and control groups (4 μM valinomycin and 100 μg/ml lysostaphin) were collected over a time-course of 20 min and filtered using a 0.22 μm pore size membrane filter. The potassium ion concentration in the filtrate samples was determined using a PerkinElmer 1100B atomic absorption spectrophotometer in flame emission mode.

### Leakage of Cellular 260 and 280 nm Absorbing Material

Leakage of nucleic acids (260 nm) and proteins (280 nm) upon treatment with the bile salts along with positive control (cells treated with 100 μg/ml lysostaphin) were monitored by measuring 260 and 280 nm absorbance in a Bio-Tek Epoch microplate spectrophotometer in UV-Visible mode. Bacterial cells were cultured and treated with selected bile salts, and samples were collected in the same manner as in the case of determination of intracellular potassium leakage except that the filtrate samples were collected over a time course of 30, 60, 90, and 120 min.

### Determination of Membrane Integrity

Changes in the membrane integrity of *S. aureus* SH1000 cells treated with various bile salts were assessed by fluorescence based confocal microscopy of dead cells with compromised cell membranes, to determine the proportion of the cells with intact cell membranes. After the treatment with bile salts, cells were subsequently incubated with a fluorescent dye mixture (component A and component B) of the LIVE/DEAD *Bac*Light bacterial viability kit (Molecular Probes, Invitrogen) according to manufacturer’s instructions for 30 min at 37°C. Images were acquired in confocal laser scanning microscope (Leica TCS SP2, Leica Microsystems, United Kingdom) and analysis was performed using the Image J analysis system.

### Scanning Electron Microscopy of *S. aureus* Treated with Various Bile Salts

*Staphylococcus aureus* SH1000 cells grown to mid-exponential phase were harvested and washed twice with 5 mM HEPES buffer (pH 7). Washed cells were resuspended at 10^8^ CFU/ml in the same buffer with or without various bile salts and incubated at 37°C for 30 min. After incubation, cells were centrifuged and pellets were fixed overnight with 2% paraformaldehyde, 2.5% glutaraldehyde fixative in 5 mM HEPES buffer at 4°C. Cell pellets were dehydrated through a graded acetone series at room temperature, critical-point dried by the CO_2_ method, and coated with gold in a sputter coater. Cells were examined and photographed with an FEI Quanta scanning electron microscope operating at 20 kV.

### Transmission Electron Microscopy of *S. aureus* Treated with Various Bile Salts

*Staphylococcus aureus* SH1000 cells for transmission electron microscopy experiments were prepared similar to that of scanning electron microscopy with following differences. After primary fixation with the 2% paraformaldehyde, 2.5% glutaraldehyde fixative in 5 mM HEPES buffer, the cells were post fixed with 1% osmium tetroxide for 1 h and dehydrated through a graded acetone series. Cells were then embedded into resin at 60°C for 48 h. After embedding, cells were sectioned into 70–80 nm thickness using an ultramicrotome (Reichert-Jung Ultracut) and mounted on formvar/carbon 200 mesh copper grids (Agar Scientific), doubly stained with 2% uranyl acetate and 0.4% lead citrate. The stained cells were viewed under a Philips CM 20 transmission electron microscope operating at 200 kV.

### Statistical Analysis

All experiments were repeated three times and data were presented as mean ± standard error of mean unless otherwise mentioned. Analysis was done in GraphPad Prism 5 software. Experimental data of membrane integrity and scanning electron microscopy measurements were analyzed by one-way ANOVA method and rest of the experimental data were analyzed by two-way ANOVA method, using Bonferroni post-test analysis. *P*-value < 0.05 was considered as statistically significant unless otherwise mentioned.

## Results

### Minimum Inhibitory Concentration

The MIC of bile salts used in this study are summarized in **Table 1**. As shown in **Table 1**, DCA showed 20 times more potent antibacterial activity than CA. CA showed at least 10 times more antibacterial activity when compared to its conjugated forms, GCA and TCA. The MIC of GCA and TCA were found to be >200 mM.

**Table 1:** Minimum inhibitory concentration (MIC) of bile salts for *S. aureus* SH1000.

Bile salt	MIC (mM)
CA	20
DCA	1
GCA	>200
TCA	>200


*CA, cholic acid; DCA, deoxycholic acid; GCA, glycocholic acid; TCA, taurocholic acid.*

### Time-Course Bacterial Viability Kinetics

**FIGURE 1:**
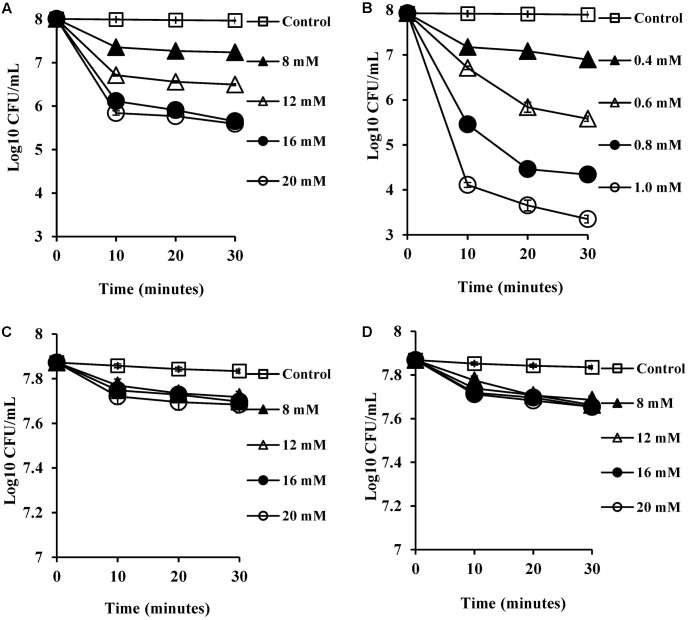
Effect of bile salts on viability of *S. aureus* SH1000. Experiments were performed at a cell density of 10^8^ CFU/ml following exposure to **(A)** CA, **(B)** DCA, **(C)** GCA, and **(D)** TCA. Data represents mean ± standard error of mean from three independent experiments.

The viability of *S. aureus* SH1000 cells upon exposure to bile salts used in this study were determined by a viable plate count method with the limit of detection at 3 Log_10_ CFU/ml. A dose-dependent reduction in viability was noticed over 30 min exposure. Almost 1 Log_10_ CFU/ml reduction (*P* < 0.01) at 8 mM, and 1.5–2 Log_10_ CFU/ml reduction in viability (*P* < 0.001) at 16 and 20 mM was found for CA (**Figure 1A**). DCA showed a similar pattern of dose-dependent reduction kinetics of viability (**Figure 1B**), but was effective at much lower concentrations. Even 0.4 mM resulted in a statistically significant reduction in viability over 30 min (*P* < 0.01). The conjugated bile salts, GCA and TCA (**Figures 1C,D**) were tested only at sub-MICs and also exhibited some dose-dependent reduction in viability. However, only a 0.2 Log_10_ CFU/ml reduction in viability (*P* < 0.01) was found at the highest concentration (20 mM) tested. The order of potency in terms of kill kinetics was thus DCA > CA > GCA = TCA.

### Bile Salts Reduce Intracellular pH

**FIGURE 2:**
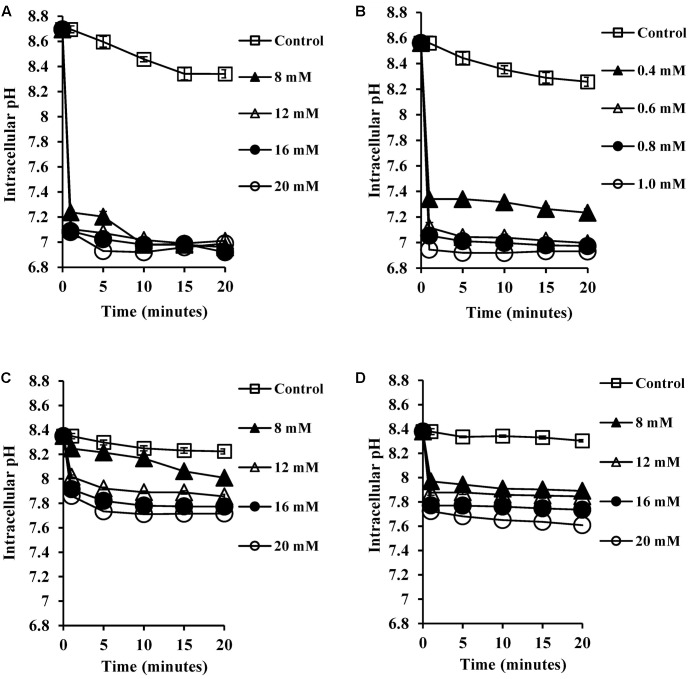
Effect of bile salts on intracellular pH of *S. aureus* SH1000. Experiments were performed at a density of 10^7^ CFU/ml cells preloaded with fluorescent probe (cFSE) and energized with 10 mM glucose following exposure to **(A)** CA, **(B)** DCA, **(C)** GCA, and **(D)** TCA. Nigericin at 4 μM was added to check the dissipation of intracellular pH. Data represents mean ± standard error of mean from three independent experiments.

SCFA, being weak acids (pKa of 4.8–5.2; Salmond et al., 1984; Russell, 1991), will exist partially in their protonated forms at pH 5.5 in the human colon. These hydrophobic molecules can traverse the plasma membrane, become deprotonated in the higher pH environment of the cytoplasm and reduce the intracellular pH (Salmond et al., 1984; Russell, 1991). Therefore, we expected that CA and DCA which are also weak acids would have similar effects in reducing intracellular pH. The intracellular pH of energized cells was monitored over a period of 20 min exposure to bile salts. The reduction in pH was found to be complete, which was confirmed by the addition of nigericin (a known ionophore which dissipates internal pH), which caused no further reduction of the internal pH of the cells. CA and DCA were found to reduce internal pH (**Figures 2A,B**) even at sub-inhibitory concentrations of 8 and 0.4 mM, respectively (*P* < 0.001). At the MIC, unconjugated bile salts caused complete reductions of the internal pH to the external pH (*P* < 0.001). Concentrations which resulted in complete dissipation of internal pH and reduction in the viability correlated closely with MICs of CA and DCA. Conjugated bile salts, GCA and TCA were also found to have a small effect on internal pH (**Figures 2C,D**) at 8 mM (*P* < 0.01). However, they were found to be significantly less effective when compared to the unconjugated bile salts. This is likely be to because conjugation makes them strong acids that are completely ionized at a neutral pH, resulting in an inability to cross cell membranes (Kurdi et al., 2006).

### Bile Salts Dissipate Transmembrane Electrical Potential

**FIGURE 3:**
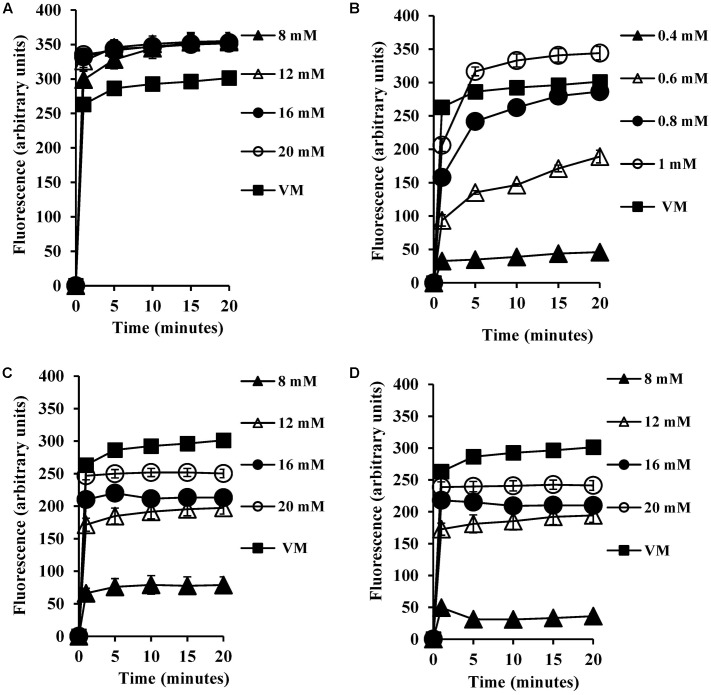
Effect of bile salts on the transmembrane electrical potential of *S. aureus* SH1000. Experiments were performed at a density of 10^7^ CFU/ml cells preloaded with the DiSC_3_ (5) dye and energized with 10 mM glucose following exposure to **(A)** CA, **(B)** DCA, **(C)** GCA, and **(D)** TCA. Dissipation of transmembrane electric potential was measured as the increase in the DiSC_3_ (5) fluorescence. VM, valinomycin at 4 μM was used as a positive control to check the dissipation of transmembrane electric potential. Data represents mean ± standard error of mean from three independent experiments.

Most microorganisms derive their energy from proton motive force consisting of a combination of an electrical potential and chemical proton gradient. We predicted that the decrease in the intracellular pH seen upon exposure to bile salts would therefore lead to alterations in the membrane potential. We therefore measured the transmembrane electrical potential using the membrane potential sensitive dye DiSC_3_ (5). The fluorescent probe DiSC_3_ (5) distributes between cells and medium depending on the cytoplasmic membrane potential. Once inside cells, it becomes concentrated and self-quenches its fluorescence. If bile salts cause cytoplasmic membrane disruption or reduce the pH gradient across the membrane, the membrane potential will be dissipated resulting in an increase in DiSC_3_ (5) fluorescence. As shown in **Figure 3A**, CA at 8 mM did indeed dissipate the membrane potential, and at 20 mM dissipation was well above that seen with the 4 μM valinomycin positive control (*P* < 0.001). The DCA dissipated membrane potential at 0.6 mM (**Figure 3B**) and at 1 mM dissipation was well above the 4 μM valinomycin positive control (*P* < 0.001). The conjugated bile salts, GCA and TCA dissipated membrane potential at 12 mM (**Figures 3C,D**; *P* < 0.01). However, these conjugated bile salts at 20 mM were unable to dissipate membrane potential completely when compared to the 4 μM valinomycin positive control, as expected given their limited effects on internal pH (Begley et al., 2005).

### Intracellular Potassium Levels

**FIGURE 4:**
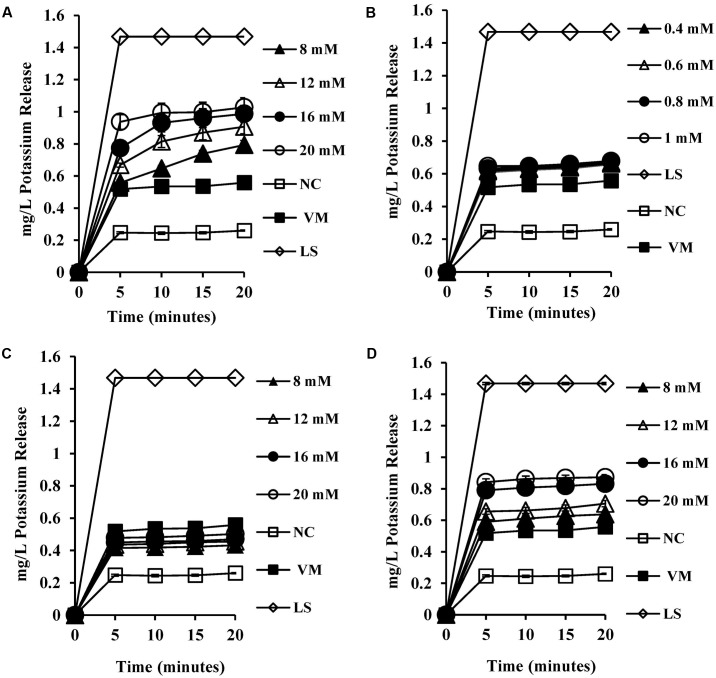
Effect of bile salts on intracellular leakage of potassium from *S. aureus* SH1000. Experiments were performed at a cell density of 10^8^ CFU/ml following exposure to **(A)** CA, **(B)** DCA, **(C)** GCA, and **(D)** TCA. NC, negative control (untreated cells); VM, valinomycin at 4 μM; LS, cells treated with 100 μg/ml lysostaphin for 30 min. Data represents mean ± standard error of mean from three independent experiments.

To test the permeability of intracellular ions caused by bile salt mediated membrane damage, we measured the amount of intracellular potassium released into HEPES buffer upon exposure to bile salts. CA exposure at 8 mM and above (**Figure 4A**) resulted in significant dose-dependent release of intracellular potassium (*P* < 0.001) when compared to both the negative control and valinomycin positive control. All the concentrations tested for DCA (**Figure 4B**) exhibited single splash type pattern of intracellular release of potassium and were statistically significant when compared to negative control (*P* < 0.01). The conjugated bile salts GCA and TCA also exhibited a dose-dependent intracellular potassium release pattern (**Figures 4C,D**) and release of potassium at 8 mM and above was statistically significant (*P* < 0.001) when compared to negative control. The release of potassium at 20 mM GCA was not significant when compared to 4 μM valinomycin control. However, TCA at 20 mM showed statistically significant release of potassium when compared to 4 μM valinomycin control (*P* < 0.01).

### Leakage of Cellular 260 and 280 nm Absorbing Material

**FIGURE 5:**
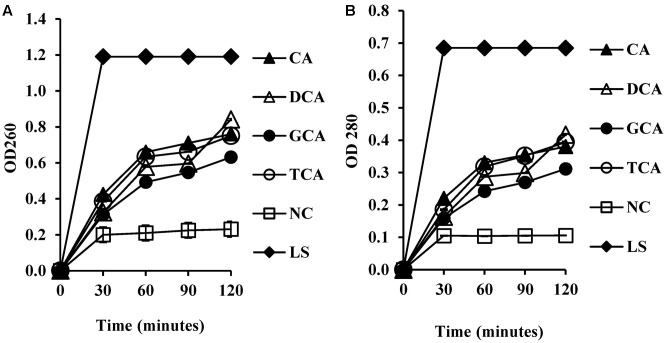
Measurement of cellular leakage of nucleic salts **(A)** and protein **(B)** from *S. aureus* SH1000 upon exposure to bile salts. Experiments were performed at a cell density of 10^8^ CFU/ml following exposure to 20 mM CA, 1 mM DCA, 20 mM GCA, and 20 mM TCA. NC, negative control (untreated cells); LS, cells treated with 100 μg/ml lysostaphin. Data represents mean ± standard error of mean from three independent experiments.

To test whether membrane damage mediated by bile salts resulted in leakage of intracellular macromolecules such as nucleic acids and proteins, UV absorbance values were measured at 260 and 280 nm following exposure of cells to bile salts. UV absorbance values at 260 and 280 nm increased significantly (**Figures 5A,B**) when compared to the negative control (*P* < 0.01). UV absorbance values increased with exposure time.

### Membrane Integrity

The membrane integrity and viability of *S. aureus* SH1000 cells upon exposure to bile salts was investigated by a fluorescent dye based confocal laser scanning electron microscopy. Image analysis revealed green fluorescence in cells with intact membrane and red fluorescence in cells with damaged membrane (Supplementary Figure 1). The unconjugated bile salts (CA and DCA) treated cells showed nearly 97% reduction in the membrane integrity (*P* < 0.001) and 97% reduction in the viability at 20 and 1 mM, respectively after 30 min exposure (**Table 2**), whereas GCA and TCA treated cells showed 50% reduction in membrane integrity and 50% reduction in the viability at 20 mM (*P* < 0.001) when compared to untreated cells (**Table 2**).

**Table 2:** Monitoring of membrane integrity of *S. aureus* SH1000 cells treated with bile salts.

Bile salt	Integrity (%)	Viability (%)
Control	91.02 ± 1.04	91.60 ± 1.05
CA	2.95 ± 0.92	2.35 ± 0.93
DCA	1.58 ± 0.53	0.97 ± 0.53
GCA	49.36 ± 1.49	49.38 ± 1.51
TCA	51.08 ± 1.95	51.12 ± 1.97


*Bacterial cells were treated at MIC of CA and DCA, and sub-MIC (20 mM) of GCA and TCA at a cell density of 10^*8*^ CFU/ml for 30 min. Data represents mean ± standard error of mean from three independent experiments. Number of cells counted = 1600. CA, cholic acid; DCA, deoxycholic acid; GCA, glycocholic acid; TCA, taurocholic acid.*

### Surface Morphology of *S. aureus* Treated with Bile Salts

**FIGURE 6:**
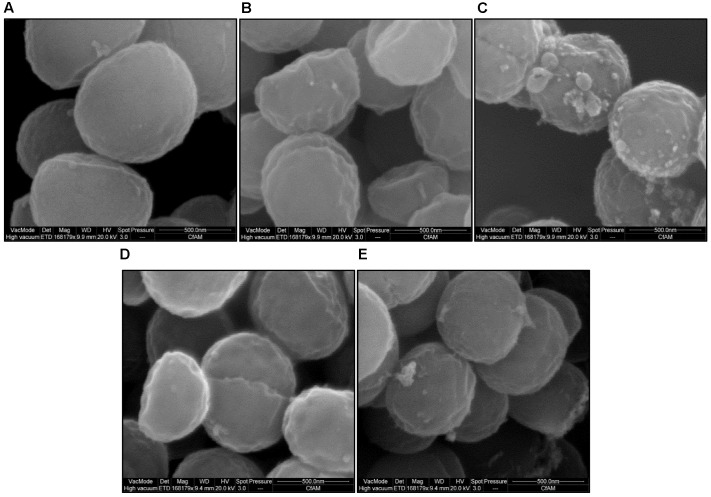
Surface morphology of *S. aureus* SH1000 in the presence of bile salts. Scanning electron microscopy was used to investigate the surface morphology of **(A)** cells untreated or treated with **(B)** 20 mM CA, **(C)** 1 mM DCA, **(D)** 20 mM GCA, and **(E)** 20 mM TCA at a density of 10^8^ CFU/ml for 30 min. Bar = 500 nm.

Scanning electron microscopy demonstrated altered surface morphology upon exposure to unconjugated bile salts (CA and DCA) at their MIC, and conjugated bile salts (GCA and TCA) at sub-MIC of 20 mM. Untreated cells had a well-defined intact spherical shape with a smooth surface morphology (**Figure 6A**). Morphological defects were severe for cells exposed to CA and DCA when compared to GCA and TCA. The cells exposed to CA and DCA had shrunken appearance and indented on the surfaces indicating possible leakage of intracellular contents. Vesicles and blebs were also present on the surface (**Figures 6B,C**). Cells exposed to GCA and TCA also had a shrunken appearance with dents on the surface (**Figures 6D,E**).

### Ultra-Structural Morphology of *S. aureus* Treated with Bile Salts

**FIGURE 7:**
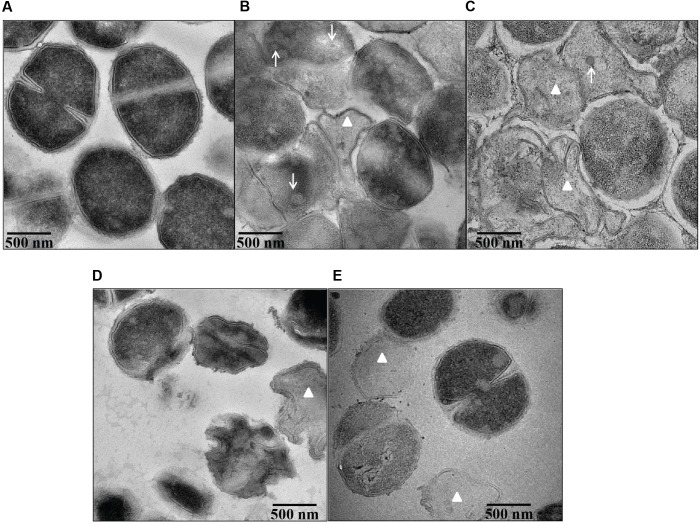
Ultra structural morphology of *S. aureus* SH1000 in the presence of bile salts. Transmission electron microscopy was used to investigate the interior morphological details of **(A)** cells untreated or treated with **(B)** 20 mM CA, **(C)** 1 mM DCA, **(D)** 20 mM GCA, and **(E)** 20 mM TCA at a density of 10^8^ CFU/ml for 30 min. Bar = 500 nm. White arrows represent mesosome like structures. White triangles represent ghost cells.

Transmission electron microscopy demonstrated pronounced ultra-structural defects in bile salts treated cells when compared untreated cells. The untreated cells revealed intact and well defined cell membrane, cell wall and midline septum with homogenous cytoplasm (**Figure 7A**). Cell wall breaks, thinning and disintegration of cell wall, cell membrane breaks, and abnormal septation were noticed in the cells exposed to all four bile salts studied (**Figures 7B–E**). Cells exposed to CA and DCA had membrane enclosed and non-membrane enclosed mesosome like structures inside the cytoplasm (Supplementary Figure 2) reflecting possible cell membrane damage and increased permeability. Many ghost cells (cells with empty cytoplasm) were also found in CA, DCA, and TCA treated cells. Cell lysis was more pronounced in CA and DCA treated cells.

## Discussion

Human bile salts in the intestine are an important facet of innate defense against enteric pathogens. They play an important role in maintaining indigenous microbiota and protection against enteric pathogens in the intestine (Sung et al., 1993; Itoh et al., 1999; Begley et al., 2005). Reduced levels of bile salts in the intestine correlate with cases of bacterial overgrowth and translocation in the small intestine, resulting in endotoxemia in cirrhotic rats (Lorenzo-Zuniga et al., 2003). Oral supplementation with bile salts in such rats can prevent small intestinal bacterial overgrowth and translocation (Lorenzo-Zuniga et al., 2003).

If one is to develop bile salts or related molecules as potential antibacterial therapy against pathogens, their mode of action needs to be established. Up till now, knowledge on bactericidal action of bile salts on *S. aureus* has been limited. Here, we propose that bile salts can kill *S. aureus* in several ways which depend on their concentration and physicochemical properties.

The accumulation of weak organic acids in the cytoplasm involves passive diffusion of the protonated form of weak acid across membranes, followed by its intracellular deprotonation and decrease in intracellular pH (Salmond et al., 1984; Russell, 1991). Bile salts, due to their structural and chemical properties, are generally considered to be weak acids and were shown to decrease intracellular pH and dissipate transmembrane potential in lactobacilli and bifidobacteria (Kurdi et al., 2006). In this study, a dose-dependent decrease in the intracellular pH in the presence of bile salts was observed. The decrease of intracellular pH was more marked with unconjugated bile salts (CA and DCA) compared to conjugated bile salts (GCA and TCA). This difference could be attributed to the higher pKa values of unconjugated bile salts (6.4 and 6.58 for CA and DCA, respectively) compared to conjugated bile salts (4 and 2 for GCA and TCA, respectively), meaning they are less dissociated at pH 7 and hence more able to cross the hydrophobic cell membrane (Carey, 1984; Cantafora et al., 1987). Conjugated bile salts are stronger acids having lower pKa values (Carey, 1984), and at physiological pH 7 are effectively fully ionized, unable to cross cell membrane unless a specific transporter is available (Begley et al., 2005). However, data presented herein also demonstrate a significant decrease in the intracellular pH at a concentration of 8 mM GCA or TCA. This concentration is close to the CMCs for GCA and TCA (7.1 and 11 mM, respectively) and it is thus possible that direct membrane damage is occurring in these cells exposed to 8 mM GCA or TCA (Roda et al., 1983; Bhairi and Mohan, 2007). Many antibacterial agents act by disrupting the cytoplasmic membrane resulting in loss of proton gradients and electrical potential across the membrane leading eventually to cell death (Nelson et al., 2009). Our results also showed a dose-dependent decrease in the transmembrane electrical potential of bile salt treated cells. Concentrations required for the reduction of transmembrane electrical potential were below the CMC of unconjugated bile salts (12 and 3 mM for CA and DCA, respectively) and above the CMC of conjugated bile salts. Thus reduction in the intracellular pH and transmembrane potential suggest that a dissipation of proton motive force is involved in bile salt mediated growth inhibition.

Our data also showed a significant (20-fold) difference between the MICs of CA and DCA, and their effect on internal pH and transmembrane electrical potential, which cannot simply be attributed to a difference in their pKa values (Carey, 1984; Cantafora et al., 1987). The more hydrophobic DCA, with two OH groups, passively flip-flops through small unilamellar vesicles at least 10 times faster than CA with three OH groups (Kamp et al., 1993). The more rapid permeation of DCA through the membrane may explain the large difference in MIC and intracellular pH reduction concentrations between CA and DCA.

The reduction in the membrane integrity demonstrated by fluorescent dye staining of *S. aureus* treated with unconjugated bile salts at their MIC, and conjugated bile salts at sub MIC of 20 mM suggested that membrane damage, which is associated with increased permeability, was occurring. This hypothesis was strengthened by detection of significant leakage of intracellular potassium from *S. aureus* cells following exposure to unconjugated and conjugated bile salts. Previous research demonstrated a similar alteration in the membrane integrity in lactobacilli and bifidobacteria, following exposure to unconjugated bile salts (Kurdi et al., 2006). Increased uptake of gentamicin in *Lactobacillus plantarum* (Elkins and Mullis, 2004) and rifaximin in enterotoxigenic *E. coli* (Darkoh et al., 2010) cells treated with bile salts suggests that besides ions, low molecular-weight metabolites might also cross the cell membrane when bile salts are present at MIC. Our study also showed a significant increase in the leakage of macromolecules such as proteins and nucleic acids in the presence of unconjugated bile salts (at MIC) and conjugated bile salts (at 20 mM), suggesting substantial cell membrane damage occurred. Bile salts at higher concentrations rapidly dissolve membrane lipids and cause dissociation of membrane proteins. This rapid solubilization effect results in leakage of cellular contents and cell death (Noh and Gilliland, 1993). Subtle effects on membrane permeability and fluidity, including altered membrane bound enzyme activities and increased transmembrane flux of divalent cations, are found at low or sub-micellar concentrations (Coleman et al., 1980; Noh and Gilliland, 1993).

Several electron microscopic studies showed cell surface damage and interior deformities in bacterial cells caused by bile salts (Ruiz et al., 2007; Merritt and Donaldson, 2009). This study demonstrated severe morphological changes, both at the surface and interior, following exposure to unconjugated bile salts at their MIC, and conjugated bile salts at 20 mM. It is likely that bile salts at sub-inhibitory concentrations exhibit bacteriostatic action through dissipation of the pH gradient and loss of transmembrane electric potential. At higher concentrations (MIC), our results suggest the bactericidal action is caused by membrane damage and leakage of intracellular contents.

Thus bile salts have multiple physiological routes by which they can inhibit *S. aureus* growth. Bile salt based derivatives, specifically a CA analog, has recently been utilized in developing novel cationic steroid antibiotics and can be used to treat topical multidrug resistant bacterial infections (Moscoso et al., 2014). This highlights their potential as novel antibacterial agents. Chemical synthesis of various analogs of bile salts utilizing specific physicochemical characteristics in their structure has begun to develop novel antibiotics to combat important bacterial pathogens (Moscoso et al., 2014).

## Author Contributions

Conceived and designed the experiments: THS and SRC. Performed the experiments: THS. Analyzed the data: THS, PAL, and SRC. Contributed to reagents/materials/analysis tools: THS, PAL, and SRC. Wrote paper: THS, PAL, and SRC. Critical revision for important intellectual content: THS, PAL, and SRC.

## Conflict of Interest Statement

The authors declare that the research was conducted in the absence of any commercial or financial relationships that could be construed as a potential conflict of interest.

## Abbreviations

CA: cholic acid
CMC: critical micelle concentration
DCA: deoxycholic acid
GCA: glycocholic acid
TCA: taurocholic acid
