# Supplementary material for: In Vitro Antibacterial Activity of Unconjugated and Conjugated Bile Salts on Staphylococcus aureus
Source: Front Microbiol. 2017 Aug 23;8:1581. doi: 10.3389/fmicb.2017.01581 (PMC5572772; doi:10.3389/fmicb.2017.01581)
Supplement: Supplementary file 1 [file Presentation_1.PDF]

Supplementary Material

In Vitro antibacterial activity of unconjugated and conjugated bile salts on *Staphylococcus aureus*

Thippeswamy H. Sannasiddappa[1, 2*], Simon R. Clarke[1] and Peter A. Lund[3]

*** Correspondence:** Thippeswamy H. Sannasiddappa; email: th526@cam.ac.uk

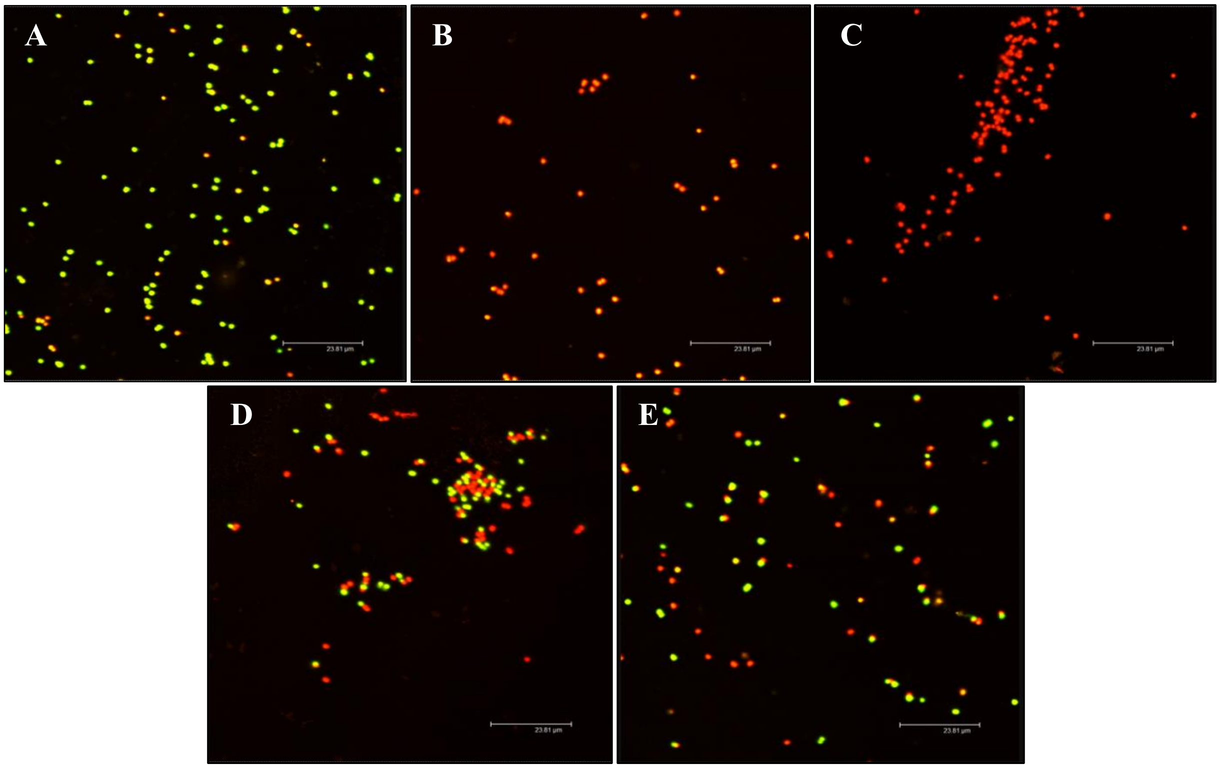

Supplementary Figure 1. Confocal scanning laser microscopy images of bile salts treated *S. aureus* SH1000 cells stained with LIVE/DEAD *Bac*Light viability stain.

Bacterial cells were exposed to no bile salts **(A)**, 20 mM CA **(B)**, 1 mM DCA **(C)**, 20 mM GCA **(D)** and 20 mM TCA **(E)** for 30 minutes. Bar = 24 µm. Bacterial cells stained fluorescent green (SYTO 9) have intact membrane, whereas bacteria stained red (propidium iodide) have damaged membranes, allowing propidium iodide to enter the cells.

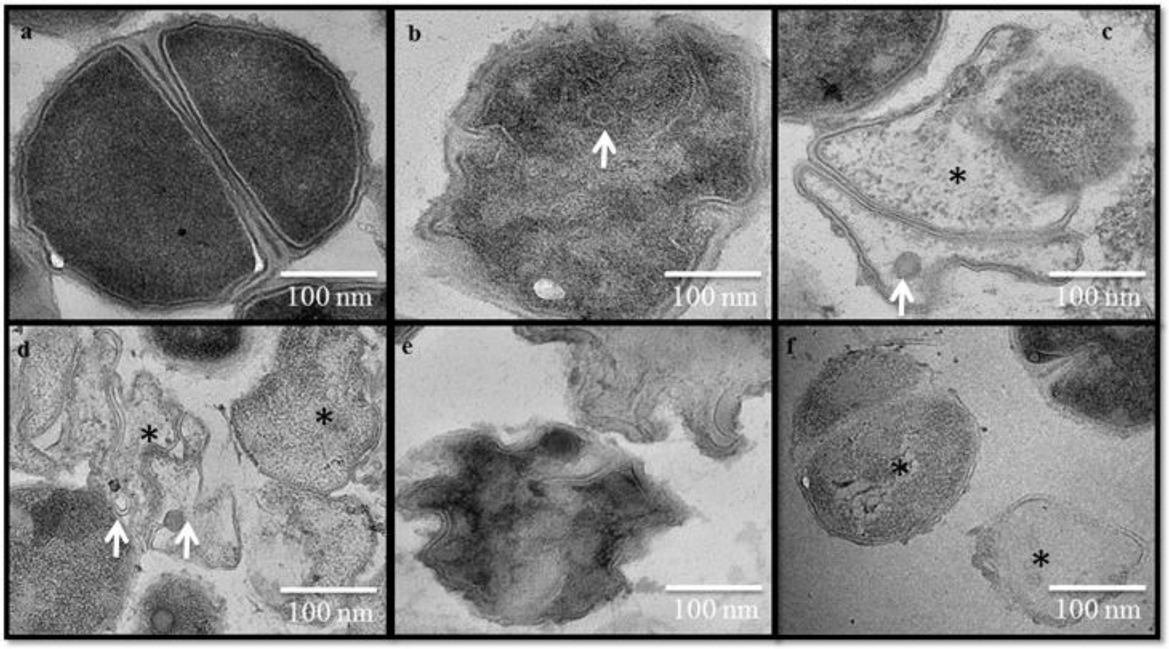

Supplementary Figure 2. **Ultra-structural morphology of *S. aureus* SH1000 in the presence of bile salts.**

Transmission electron microscopy was used to investigate the interior morphological details of cells untreated **(a)** or treated with 20 mM CA **(b)**, 1mM DCA **(c and d)**, 20 mM GCA **(e)** and 20 mM TCA **(f)** at a density of 10^8 CFU/ml for 30 minutes. Bar = 100 nm. White arrows represent mesosome like structures. Black asterisks represent ghost cells.
